# Lymphatic Leukaemia in Acute Infection after Splenectomy

**Published:** 1915-03

**Authors:** 


**Lymphatic Leukaemia in Acute Infection After Splenectomy.** James Jackson and Wm. David Smith, Boston, Boston M. & S. Jour., Jan. 28, 1915. Male, splenectomy for traumatic rupture, in 1909 at age of 17; acute tonsillar infection in 1913 at age of 21. Shortly after splenectomy, the reds were 4,600,000; whites 7,800; Hb. 70%; differential count: neutrophile poly nuclears 58%, small lymphocytes 22.5%, large lymphocytes 2.5%, transitionals 10%, easinophiles 6%, mast cells 1%. About a week after the tonsillar infection began, the whites numbered 40,000 and the respective leucocytes 5%, 36%, 59%, last three types not found. June 8, the whites had declined to 7000 and the patient was apparently well. April 15, 1914, there was no leucocytosis, red cells normal, polymorphous cells 75%, small lymphocytes 13%, large 6%, osinophiles 6%, platelets abundant Note: The interest in this case attaches mainly to the use of the terms leukaemia and leucocytosis. While there is positive scientific basis at present for holding that there is no such thing as a mild and curable leucocythaemia, there are good practical reasons for limiting the term to very large proportions of white cells, say 100,000 until influenced by treatment, to demand marked qualitative abnormalities and to exclude inflammatory reactions, especially when they subside in a short time).

